# Pathologic and immunohistochemical prognostic markers in residual triple-negative breast cancer after neoadjuvant chemotherapy

**DOI:** 10.3389/fonc.2023.1309890

**Published:** 2024-01-10

**Authors:** Silvia Mihaela Ilie, Nathalie Briot, Guillaume Constatin, Alis Ilie, Francoise Beltjens, Sylvain Ladoire, Isabelle Desmoulins, Audrey Hennequin, Aurelie Bertaut, Charles Coutant, Sylvain Causeret, Niama Ghozali, Bruno Coudert, Laurent Arnould

**Affiliations:** ^1^ Department of Medical Oncology, Georges Francois Leclerc Cancer Centre, Dijon, France; ^2^ Department of Biostatistics Georges Francois Leclerc Cancer Centre, Dijon, France; ^3^ Cancer Biology Research Platform, Centre Georges Francois Leclerc, Dijon, France; ^4^ Department of Bio-pathology, Georges Francois Leclerc Cancer Centre, Dijon, France; ^5^ Surgery Department Georges Francois Leclerc Cancer Centre, Dijon, France; ^6^ Department of Medical Oncology, University Hospital Mohammed VI, Tangier, Morocco

**Keywords:** neoadjuvant chemotherapy, residual disease, triple-negative breast cancer, prognostic biomarkers, immunohistochemical marker

## Abstract

**Background:**

The persistence of residual tumour after neoadjuvant chemotherapy (NAC) in localised triple-negative breast cancer (TNBC) is known to have a negative prognostic value. However, different degrees of expression of some immunohistochemical markers may correlate with different prognoses.

**Methods:**

The expression of biomarkers with a known prognostic value, i.e., cytokeratin 5/6 (CK5/6), androgen receptor (AR), epidermal growth factor receptor (EGFR) proliferation-related nuclear antigen Ki-67, human epidermal growth factor receptor 2 (HER2), protein 53 (p53), forkhead box protein 3 (FOXP3), and cluster differentiation 8 (CD8), was analysed by immunohistochemistry in 111 samples after NAC in non-metastatic TNBC patients addressed to Georges-François Leclerc Cancer Centre Dijon, France. Clinical and pathological variables were retrospectively collected. Cox regression was used to identify immunohistochemical (IHC) and clinicopathological predictors of event-free survival (EFS) (relapse or death).

**Results:**

Median age was 50.4 years (range 25.6–88.3), 55.9% (n = 62) were non-menopausal, 70 (63.1%) had stage IIA–IIB disease. NAC was mostly sequential anthracycline-taxanes (72.1%), and surgical intervention was principally conservative (51.3%). We found 65.7% ypT1, 47.2% lymph node involvement (ypN+), and 29.4% lymphovascular invasion (LVI). Most residual tumours were EGFR >110 (H-score) (60.5%, n = 66), AR ≥4% (53.2%, n = 58), p53-positive mutated (52.7%, n = 58), CD8 ≥26 (58.1%, n = 61), FOXP3 ≥7 (51.4%, n = 54), more than half in the stroma, and 52.3% (n = 58) HER2 score 0. After a median follow-up of 80.8 months, 48.6% had relapsed. Median EFS was 62.3 months (95% CI, 37.2–not reached (NR)). Factors independently associated with poor EFS were AR-low (p = 0.002), ypN+ (p < 0.001), and LVI (p = 0.001). Factors associated with lower overall survival (OS) were EGFR-low (p = 0.041), Ki-67 high (p = 0.024), and ypN+ (p < 0.001).

**Conclusion:**

Post-NAC residual disease in TNBC showed biomarkers specific to a basal-like subtype and markers of lymphocyte infiltration mostly present in the stroma. Prognostic markers for EFS were AR, LVI, and ypN and warrant further validation in a prognostic model.

## Introduction

Triple-negative breast cancer (TNBC) is defined by the absence of immunohistochemical expression of hormone receptors (estrogen, progesterone) and absent or very low expression of human epidermal growth factor receptor 2 (HER2) (1, 2). TNBC represents approximately 15% of breast cancers and remains the most aggressive phenotype (3). Residual disease (RD) after neoadjuvant chemotherapy (NAC) in localised TNBC is a negative prognostic factor in terms of relapse rate and disease-free survival (DFS) (4–6). For this reason, in the absence of pathologic complete response (pCR), breast cancer guidelines recommend systemic treatments post-surgery (7, 8).

Since the last positive trial proposing capecitabine in case of invasive RD in aggressive localised breast cancer after NAC (9), a panoply of post-neoadjuvant treatments have emerged, such as olaparib in germinal breast cancer (gBRCA) gene mutated cases or pembrolizumab (10, 11), to target more specifically the remaining tumour, as a demonstration of the need to reduce the risk of recurrence in these cases.

Localised TNBC is widely analysed by gene sequencing in chemo-naive primary tumour or in RD, and several prognostic scores have been proposed, but this approach is too expensive to be feasible in everyday clinical practice (12–14).

Several biomarkers assessable by immunohistochemistry have been analysed by other teams for their prognostic role in TNBC, regardless of whether the tumour was primary or residual. These biomarkers include epidermal growth factor receptor (EGFR), cytokeratin 5/6 (CK5/6), or proliferation-related nuclear antigen Ki-67 (15), as well as immunological markers, such as cluster differentiation 8 (CD8) and forkhead box protein 3 (FOXP3) expression, which are specifics to cytotoxic and regulatory T lymphocytes, respectively (16).

We selected some classical immunohistochemical biomarkers, with a view to analysing their prognostic role for event-free survival (EFS) (relapse or all-cause death) in residual tumour after NAC in localised TNBC.

## Materials and methods

### Data source and study

**Figure 1:**
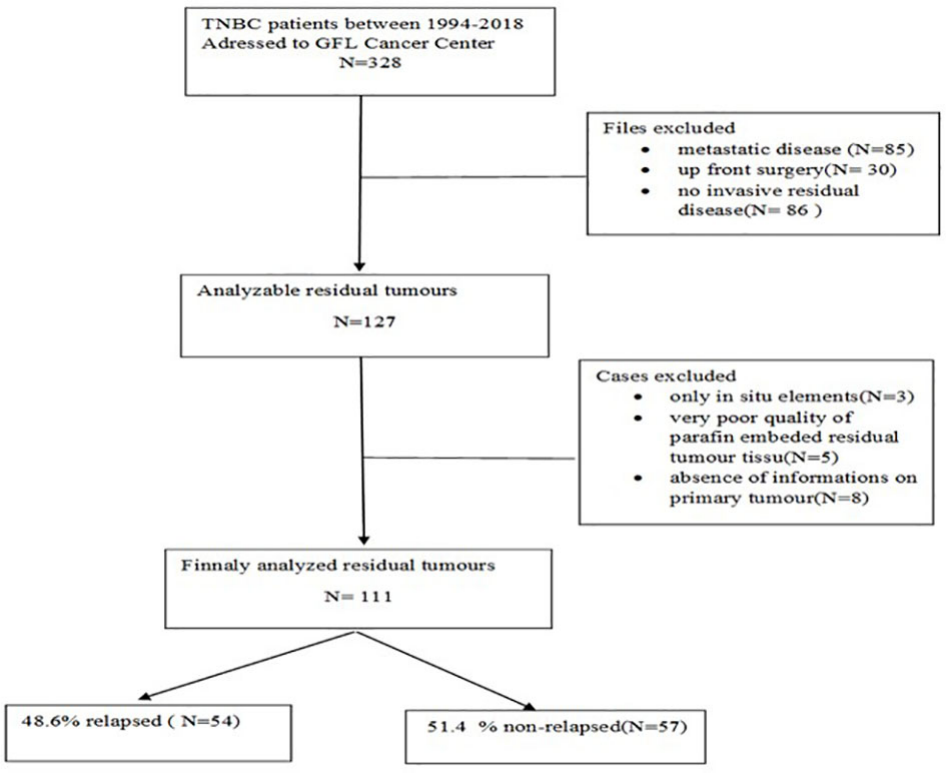
Consort Diagramme of the study.

All patients with TNBC treated by NAC followed by surgery between 1994 and 2018 in the Georges-François Leclerc Cancer Center in Dijon, France, who displayed RD were included in this study (see CONSORT diagram in **Figure 1**). The study was declared on ClinicalTrials.gov under the identifier NCT04031612 and was carried out in accordance with the Helsinki Declaration and approved by CNIL (French National Commission for Data Privacy).

The inclusion criteria were as follows: adult women with unilateral localised TNBC according to the AJCC 8th edition (17) with a threshold of <10% for estrogen receptor (ER) and progesterone receptor (PgR) staining according to the definition in France (18) and according to the American Society of Clinical Oncology/College of American Pathologists (ASCO/CAP) methods (2) and without HER2 overexpression, according to the CAP/ASCO definition from 2007 to 2018 if applicable, and otherwise according to local practice (19, 20).

We retrospectively analysed pathological parameters of RD, namely, the number and diameter of foci of invasive carcinoma, the number and type of lymph node involvement, the presence of *in situ* components, and the presence of lymphovascular or perineural involvement.

### Immunohistochemistry

Tumour grade was classed according to the Scarff-Bloom-Richardson (SBR) grading system (21). CK5/6, EGFR, androgen receptor (AR), Ki-67, protein 53 (p53), FOXP3, CD8, and HER2 expression was assessed by immunohistochemistry on the residual paraffin-embedded surgical specimen following the REMARK guidelines (22). Briefly, 4–5-µm sections were stained with hematoxylin–eosin solution to identify and characterise residual invasive carcinoma, and IHC staining was subsequently performed. The antibody clones used and the immunohistochemistry protocol are indicated in the **Supplementary Material** for each biomarker (**Supplementary Material 1**).

CK5/6 was assessed as the percentage of cytoplasmic staining, whatever the intensity (23). EGFR expression was quantified by the H score, which comprises the percentage of positive membranous staining from negative to slightly positive (1+), moderately positive (2+), and strongly positive (3+), yielding values from 0 to 300 (all cells strongly positive) (24). AR was quantified as the percentage of nuclear staining, whatever the intensity (25).

For p53 positivity, we established two categories, namely, “very strong positive,” corresponding to missense mutation, and “moderate positive,” corresponding to non-mutated status, with normal p53 synthesis and no stain corresponding to a deletion mutation (26).

CD8 and FOXP3 nuclear staining were counted as absolute numbers in adjacent stromal and intratumoural compartments. We calculated the median value of five areas (27).

Cell proliferation was assessed by nuclear staining in at least 500 tumour cells in at least three representative fields using the corresponding antibody for Ki-67 and using the online software recommended by the French Association of Quality Assurance in Pathologic Anatomy and Cytology (AFAQAP): https://www.afaqap.fr/index-ki67/.

HER2 was assessed and quantified by IHC according to the ASCO/CAP 2018 guidelines (28).

IHC analysis was performed in our in-house laboratory and evaluated by two physicians. In case of disagreement, the fields were reexamined by high-power ×40 lens until a consensus was reached.

### Statistical analysis

Categorical variables are described as number and percentage and continuous variables as mean ± standard deviation (SD) or median and interquartile range (IQR). Continuous variables were compared between groups using the Student’s t-test in case of normally distributed variables or the Wilcoxon test in case of non-normal distribution. The Shapiro–Wilk test was used to check the normality of the distribution. Categorical variables were compared using the chi-square or Fisher’s exact test, as appropriate. Tests were two-sided, and the threshold of significance was fixed at 5%. The median follow-up was calculated according to the reverse Kaplan–Meier (KM) method. Survival rates and median survival times with their associated 95% confidence interval (CI) were determined using the KM method. EFS was defined as the time in months between the date of breast cancer diagnosis and the first recurrence of either locoregional or distant metastasis or death. Overall survival (OS) was defined as the time in months between diagnosis and death from any cause or last follow-up. Survival curves were compared using the log-rank test. Univariate and multivariate Cox regression analyses were performed to determine independent predictive factors of survival. All variables with a p-value <0.20 and with <20% missing data were included in the multivariable model, which was adjusted for age. Correlations between eligible variables were tested. The threshold for retention in the final model was p < 0.05.

To determine the appropriate threshold for each biomarker, we used Cut Off finder web application (https://molpathoheidelberg.shinyapps.io/CutoffFinder_v1/). This method fits Cox proportional hazard models to the dichotomised variable and the survival variable. The optimal cutoff is defined as the point with the most significant split (log-rank test) (29). If no threshold was significant at 5% for a biomarker (or if the groups were too different in size), we used the median as the threshold. All statistical analyses were performed using SAS version 9.4 (SAS Institute Inc., Cary, NC, USA).

## Results

### Patients and treatment characteristics

Among 111 cases analysed (**Figure 1**), the median age was 50.4 years (25.6–88.3), 55.9% (n = 62) were non-menopausal, the majority (63.1%, n = 70) had stage IIA–IIB disease, mostly involving the left breast 60.4% (n = 64) and external quadrant 55.9% (n = 62). Radiologically, the tumour was principally unicentric and unifocal (88.3%, n = 98), and the main histopathological type on biopsy was ductal carcinoma (n = 107, 96.4%). A total of 71.6% (n = 78) were grade III and mostly ER-negative (92.8%, n = 103). HER2 expression was scored 0 in 54.5% (n = 60). Mean Ki-67 in the biopsy specimen was 42% ± 27.6%.

NAC was mostly sequential anthracycline-taxanes (72.1%, n = 80), and the surgical intervention was mainly conservative (51.3%, n = 57).

Forty-three patients (40.2%) had a family history of cancer, but germinal BRCA1/2 mutation was found in only 9 cases (56.3%) of the 16 in whom this analysis was performed.

Regarding the pathologic characteristics of RD, the mean diameter of the invasive component was 21.4 ± 11.1 mm, 66.7% (n = 74) were unifocal, the tumour pathological stage was predominantly ypT1c (29.7%, n = 33). Postoperative lymph node involvement (ypN+) was found in 52 cases (47.2%), including ypN3a in 11.8% (n = 13) and 29.4% had lymphovascular invasion (LVI) (n = 31).

A total of 108 patients (97.3%) received post-neoadjuvant treatment, of which 74.1% (n = 80) was radiotherapy.

After a median follow-up of 80.8 months (9.3–216.6), relapse was observed in 48.6% (n = 54), of whom 29.6% (n = 16) had visceral metastasis; the median time to progression was 25 months (9–87.6), and the death rate was 40.5% (n = 45). More than half (63.5%, n = 40) of the relapsed patients received second-line systemic treatment (**Table 1**).

**Table 1:** Baseline characteristics.

Variable	Number (111)/ Percentage (100%)
Age at diagnosis	
median range	50.4 (25.6-88.3)/100
Menopausal status	
Premenopausal Postmenopausal	62(55.9) 49(44.1)
Germinal BRCA1/2 mutation*	
Yes No	9(56.3) 7(43.8)
cT stage	
T1 T2 T3 T4	2(1.8) 62(55.9) 24(21.6) 23(20.7)
cN	
N0 N1 N2a/b N3a/b/c	43(38.7) 53(52.3) 6(5.4) 4(3.6)
cTNM	
IIA/IIB IIIA/IIIB/IIIC	70(63.1) 41(36.9)
Laterality*	
Right Left	42(39.6) 64(60.4)
Quadrant	
Internal External Central Multicentric	35(31.5) 62(55.9) 12(10.8) 2(1.8)
Radiological tumour characteristics	
Unicentric unifocal Unicentric multifocal Multicentric	98(88.3) 9(8.1) 4(3.6)
HP subtype	
Ductal Other (apocrine, metaplastic)	107(96.4) 4(3.6)
SBR grading*	
I II III	2(1.8) 29(26.6) 78(71.6)
Estrogen receptors	
0% 1%–10%	103(92.8) 8(7.2)
Progesterone receptors	
0% 1%–10%	106(95.5) 5(4.5)
HER2	
Score 0 Score 1+ Score 2+, ISH negative	60(54.5) 25(22.7) 25(22.7)
Ki-67 on biopsy specimen*	105(94.6)
Median range	43 (1–90)
NAC chemotherapy	
A-T containing q3w A-T q2 w Platinum-based A only	80(72.1) 2(1.8) 1(0.9) 28(25.2)
Type of surgery	
Conservatory Conservatory tumour ALND Radical tumours SLNB Radical	12(10.8) 45(40.5) 5(4.5) 49(44.1)
Pathologic diameter of invasive component (total diameter) Mean ± SD, range	21.4 ± 11.1 (4-35)
ypT	
T0 T1a T1b T1c T2 Tx	1(0.9) 28(25.2) 12(10.8) 33(29.7) 31(27.9) 6(5.4)
ypN*	
N0 N1a N2a N3a	58(52.7) 26(23.6) 13(11.8) 13(11.8)
LVI*	
Yes No	32(29.4) 77(70.6)
Post-neoadjuvant treatment*	
No Radiotherapy Chemotherapy Multimodal	3(2.7) 80(74.1) 2(1.8) 26(24.1)
Relapse	
Yes No	54(48.6) 57(51.4)
Locoregional relapse	
Yes No	30(37) 81(63)
Metastatic relapse	
No Non-visceral Visceral CNS	77(69.4) 11(9.9) 16(14.4) 7(6.3)
PFS	
Median, range	25 (9-87.6)

BRCA, BReast Cancer; c, clinical; T, tumours; N, lymph node; M, metastasis; HP, histopathological; SBR, Scarff-Bloom-Richardson; HER2, human epidermal growth factor receptor 2; ISH, immunofluorescence; Ki-67, proliferation-related nuclear antigen; NAC, neoadjuvant chemotherapy; A, anthracyclines; T, taxanes; q2-3w, once every 2–3 weeks; ALND, axillary lymph node dissection; SLNB, sentinel lymph node biopsy; SD, standard deviation; ypT, post-therapeutic pathologic tumour stage; ypN, post-therapeutic pathologic lymph node stage; LVI, lymphovascular invasion; CNS, central nervous system; PFS, progression-free survival; *, variables containing missing values.

### Frequency of expression of biomarkers and their individual prognostic significance

RD was mostly HER2 score 0 (52.3%); Ki-67 low, <43% (50.5%); CK5/6 low, ≤29% (50.5%); EGFR high, ≥100 H score (60.5%); AR high, ≥4% (53.2%); CD8 high, ≥26 (60%); and FOXP3 high, ≥7 (56.2%). CD8 was found mainly in the stroma, median 23 (0–99), while in the intratumoural compartment, representation was lower, median 3 (0–67). Similarly, for FOXP3, representation was higher in the stroma, median 7 (0–46), and very low in the tumour residue, median 0 (0–16). p53-positive strong intensity (by convention, corresponding to missense mutated TP53 status) was observed in 52.7% (n = 58) and negative (by convention, corresponding to deletion mutated TP53 status) in 16.4% (n = 18).

The frequency of expression of each biomarker in RD is displayed in **Table 2**.

**Table 2:** Biomarker characteristics in residual disease.

Biomarker	Number	Percentage
Ki-67		
Median, range ≤43 >43	43 (1-90) 53 52	50.5 49.5
HER2		
Score 0 Score 1 Score 2+	58 18 35	52.3 16.2 31.5
EGFR		
Median, range Cutoff EFS <110 ≥110	104 (1-256) 43 66	39.5 60.5
CK5/6		
Median, range ≤29 >29	29 (0-100) 53 52	50.5 49.5
AR		
Median, range Cutoff EFS <4 ≥4	2 (0-100) 51 58	46.8 53.2
p53		
Negative Positive non-mutated Positive mutated	18 34 58	16.4 30.9 52.7
CD8total		
Cutoff EFS		
<26 ≥26	44 61	41.9 58.1
CD8 intratumoural		
Mean ± SD, median (range)	7.2 ± 11.8, 3 (0-67)	
CD8 stromal		
Mean ± SD, median (range)	29.3 ± 23, 23 (0-99)	
FOXP3total		
Cutoff EFS		
<7 ≥7	51 54	48.6 51.4
FOXP3 intratumoura		
Mean ± SD, median (range)	1 ± 2.1, 0 (0-16)	
FOXP3 stromal		
Mean ± SD, median (range)	8.5 ± 8.2, 7 (0-46)	

Ki-67, proliferation-related nuclear antigen; HER2, human epidermal growth factor receptor 2; EGFR, epidermal growth factor receptor; CK5/6, cytokeratin 5/6; AR, androgen receptor; p53, protein 53; CD8, cluster of differentiation 8; SD, standard deviation; EFS, event-free survival; FOXP3, forkhead box protein 3.

**Figure 2:**
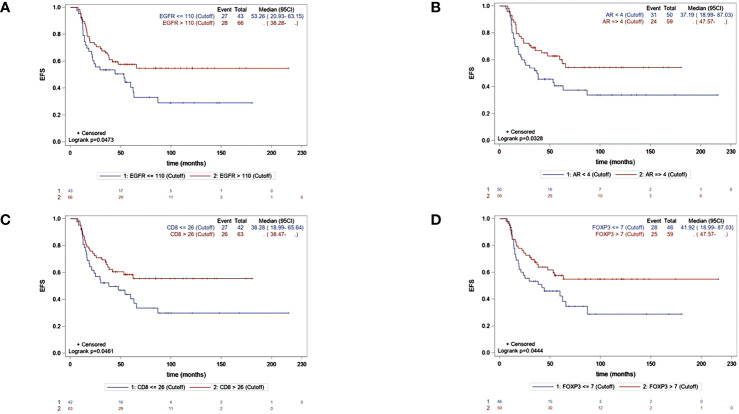
KM curves for EFS accordingly significant biomakers expression :EGFR **(A)**, AR **(B)**, CD8 **(C)**, FOXP3 **(D)**.

**Figure 3:**
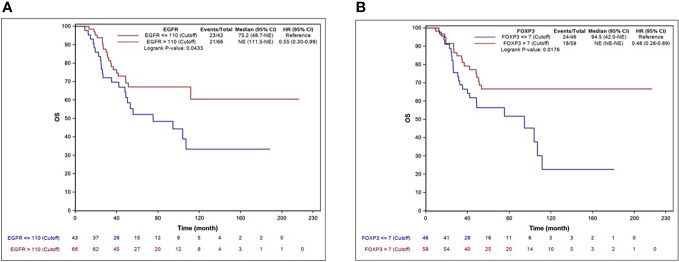
KM curves for OS according significant biomakers expression. EGFR **(A)**, FOXP3 **(B)**.

The median EFS was 62.3 months (95% CI, 37.2–NR), and the median OS was 111.5 months (95% CI 94.5–NR). By univariate Cox regression analysis, the biomarkers significantly associated with longer EFS were EGFR, AR, CD8, and FOXP3. The factors associated with OS were EGFR and FOXP3. Ki-67 >43% was significantly associated with shorter OS. The independent predictive factors for EFS identified by multivariate analysis are shown in **Table 3**. KM EFS curves showed better outcomes associated with CD8+ [hazard ratio (HR) 0.58, 95% CI 0.34–1, p = 0.0461], FOXP3 (HR 0.58, 95% CI 0.34–0.99, p = 0.044), EGFR >110 (HR 0.59, 95% CI 0.35–1, p = 0.0473), and AR ≥4 (HR 0.56, 95% CI 0.33–0.96, p = 0.0328). Regarding OS, the biomarkers for which a high level of expression was associated with improved survival were EGFR >110 (HR 0.55, 95% CI 0.3–0.99, p = 0.043) and FOXP3 >7 (HR 0.48, 95% CI 0.26–0.89, p = 0.0176). The KM curves for EFS and OS for each biomarker are illustrated in **Figures 2**, **3**.

**Table 3:** Univariate and multivariate Cox regression for EFS and OS.

Variable	Univariate EFS (95% CI)	p	Multivariate EFS (95% CI)	p	Univariate OS (95% CI)	p	Multivariate OS (95% CI)	p
Age at diagnosis >50vs. ≤50	1.84 (1.08-3.15)	0.025	1.58 (0.83-2.99)	0.16	2.37 (1.29-4.36)	0.006	2.44 (1.23-4.84)	0.01
Menopausal status Yes vs. No	1.68 (0.99-2.85)	0.053			2.37 (1.31-4.31)	0.005		
cN N1vs. N0 N2-3vs. N0	2.18 (1.19-3.98) 1.53 (0.56-4.21)	0.039						
ypN N1vs. N0 N2-3 vs. N0	2.38 (1.2-4.72) 6.85 (3.61-13)	<0.001	1.94 (0.93-4.08) 5.95 (2.79-12.7)	<0.001	2.55 (1.18-5.55) 5.99 (2.92-12.3)	<0.001	2.07 (0.92-4.68) 4.28 (2.01-9.12)	<0.001
LVI Yes vs. No	3.37 (1.97-5.78)	<0.001	2.82 (1.51-5.25)	0.001	3.31 (1.83-6.02)	<0.001		
Ki-67 >43vs. ≤43					1.84 (1-3.41)	0.05	2.15 (1.1-4.2)	0.024
EGFR >110vs. ≤110	0.59 (0.35-1)	0.05			0.55 (0.3-0.99)	0.047	0.52 (0.28-0.97)	0.041
AR ≥4%vs.<4%	0.56 (0.33-0.96)	0.035	0.29 (0.16-0.54)	<0.001				
CD8 >26vs.≤26	0.58 (0.34-1)	0.049						
FOXP3 >7vs.≤7	0.58 (0.34-0.99)	0.047			0.48 (0.25-0.89)	0.02		

c, clinical; N, lymph node; yp, posttreatment pathological stage; LVI, lymphovascular invasion; Ki-67, proliferation-related nuclear antigen; EGFR, epidermal growth factor receptor; AR, androgen receptor; CD8, cluster of differentiation 8; FOXP3, forkhead box protein 3; EFS, event free survival; OS, overall survival.

## Discussion

In the present study, we investigated the prognostic role of a panel of immunohistochemical biomarkers whose clinical significance in breast cancer, especially the TNBC phenotype, has previously been studied. We sought to identify candidates that, in a subsequent analysis, extended to a larger number of cases, could be evaluated to create a multivariate prognostic score for RD.

### Residual disease and biomarker expression

**Figure 4:**
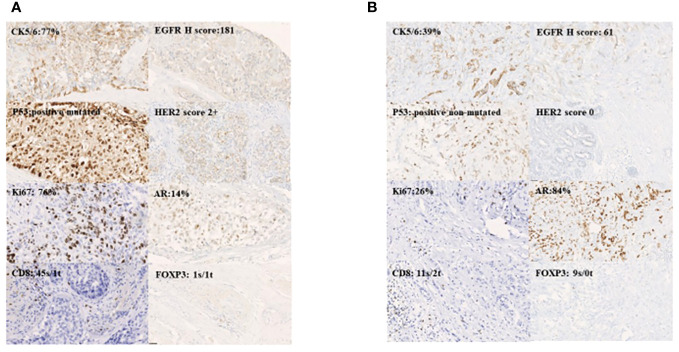
Representative photomicrographs for biomakers expression magnification 20X:basal-like **(A)** and luminal AR+ **(B)**.

In our immunohistochemical analysis, the tumour residue was rich in EGFR and intensely p53-positive, the last one corresponding to TP53 missense mutation status. HER2 was mainly scored 0, corresponding to the initial status, while the Ki-67 value was intermediate for a triple-negative phenotype, CK5/6 was mainly low ≤29% and AR was high ≥4%. Representative photomicrographs for biomarker expression (×20 magnification) are shown in **Figures 4A, B**.

Our cutoff for Ki-67 of 43% is consistent with the results in the literature (30). In a recent meta-analysis that included mainly Asian studies (35 studies totalling 7,716 patients), and in which assessments were mainly performed in the chemo-naive TNBC tumour specimen, the cutoff of Ki-67 that was significant for DFS (HR 2.3, 95% CI 1.54–3.44, p < 0.001) and for OS (HR 2.95, 95% CI 1.67–5.19, p < 0.001) was 40% (31).

HER2 score 0 was found in 52.3% of the cases analysed, approaching the values found in the literature in triple-negative nonmetastatic breast cancer in general (32, 33), less evaluated in a targeted manner in the residual tumour (34).

EGFR overexpression is found in 50% of TNBC patients and in up to 90% in the basal-like subtype (35) for which it is also used as a surrogate (36).

CK5/6 is known as a marker of basal-like sub-phenotype and the percentages of positivity reported in triple-negative cancers are approximately 50% consistent with our result even though most analyses considered positive any identified staining while we set the cutoff of positivity according to the median percentage of positive cells (29%).

In our analysis, p53 was found positive in 83.6% (n = 92) of which 52.7% were positive-mutated. In the literature, p53 expression is found in more than half of the TNBC cases, regardless of the method of assessment, and correlates with EGFR overexpression (37). It is always associated with poor prognosis (38, 39). Some studies have reported that positivity could mean the presence of a poor-quality protein in the cytoplasm and thus correlates with an aggressive phenotype (38) or produced in excess as a compensatory mechanism of tumour DNA repair and to be a good prognostic factor (40).

Regarding the scoring system and cutoff for AR, in a 2020 meta-analysis, it was found that the majority of studies evaluated the expression of AR by the percentage of positive cells, of which 44.44% (12 studies) used a low cutoff value (0% or 1% or 7.5%), 37.04% (10 studies) used a higher cutoff value (10 or 45%), and only five studies (18.25%) used other methods such as Allred, which took into account the intensity and the percentage of positive cells (41).

A profile combining Ki-67 low, CK5/6 low, and AR positive status, mainly found in RD in our cases, could indicate selection by NAC of a luminal androgen receptor (LAR) phenotype containing cells that are less chemosensitive (13).

We choose CD8 and FOXP3 as surrogate markers for tumour-infiltrating lymphocytes (TILs) whose prognostic role is already known (42), since the lymphocyte infiltrate is heterogeneous, containing both helper and cytotoxic lymphocytes and regulatory-inhibitory lymphocytes (43), and as recent studies have shown direct correlations between TILs, programmed death-1 ligand (PD-L1), CD8, and FOXP3 in early TNBC (44).

In more than 50% of cases, the lymphocytic infiltrate was predominantly found in the stroma and was balanced in terms of CD8+ and FOXP3+ representation. CD8 and FOXP3 high were found to be associated with favourable prognosis for EFS, albeit only by univariate analysis.

Abundant CD8+ lymphocytic infiltrate is known to be associated with positive prognosis in ER-negative breast cancer (45). Other authors have reported that both peritumoural CD8+ and FOXP3+ lymphocytic infiltrates are associated with a good outcome (16), mainly in receptor-negative early breast cancer (46). In a recent study that aimed to determine the prognostic role of CD8, FOXP3, and PD-L1 expression in early-stage TNBC, the recognised marker of regulatory T lymphocytes (Tregs), FOXP3 (47) was found to correlate with better survival (HR 0.48, 95% CI 0.28–0.80, p = 0.004) for a cutoff of 57 (44). As our CD8/FOXP3 ratio was in favour of CD8, it is concordant with the results of another study published in 2015 and reporting that a high ratio predicts a good prognosis (48).

### Individual prognostic role

In our analysis, the only biomarker whose expression had a protective effect against relapse was overexpression of androgenic receptors. AR expression has a controversial prognostic value in early TNBC. Several large retrospectives studies and meta-analyses have shown an association with improved DFS and OS (49–51), while others have reported an increase in mortality (52, 53). Few studies to date have evaluated the prognostic role of AR in residual tumour. In one study that evaluated the change in AR from primary to residual tumour after NAC, in 71 cases of localised TNBC, the authors found that AR loss was associated with a favourable prognosis, notably in terms of 5-year distant free survival rate (61.6%, 95% CI 44.26–79.14) vs. 25% (95% CI 3.94–87.21) (p = 0.01) in the groups with vs. without AR loss, respectively (54).

Among the pathological factors, LVI and residual lymph node involvement were found to be predictors of relapse. These are classically recognised as negative prognostic factors in localised breast cancer and are also found in specific analyses in the triple-negative phenotype. Accordingly, in a study by Kennedy et al. (55), in 108 patients with triple-negative residual tumour who had received NAC, the factors that were independent predictors of metastatic DFS were node positivity (HR 3.08, 95% CI 1.54–6.14, p = 0.001) and LVI (HR 1.91, 95% CI 1.07–3.43, p = 0.30). By summing negative prognostic factors including the presence of residual tumour with the addition of initial multifocal status, a prognostic model was developed based on the impact on survival, as follows: 0 factor, 5-year freedom from distant metastasis (FFDM) rate of 76%, and 4 factors, 0% of FFDM (55).

For OS, the biomarkers directly associated with the risk of death were the classic Ki-67 high and, interestingly, EGFR low status. Also, residual lymph node involvement emerged once again as a negative prognostic factor.

As regard to EGFR, in our study, we found that, according to our cutoff, receptor’s overexpression was an independent positive prognostic factor for OS. In the literature, results are conflicting regarding EGFR overexpression, with meta-analysis and retrospective analysis showing it to be an adverse prognostic factor (36, 56), while other reports failed to replicate this (57). Accordingly, in one study of a representative triple-negative European population, EGFR >50% assessed in the primary tumour (n = 284) was associated with a 2-fold increase in the risk of recurrence and death (HR for 4-year DFS 2.39, 95% CI 1.32–4.34, p = 0.004 and for OS, HR 2.34, 95% CI 1.2–4.9) (58). In an Asian study (n = 287), for EGFR >10%, 5-year DFS was significantly lower (69% vs. 83.8%, p = 0.011) as was OS (79.5% vs. 88.9%) in patients with EGFR <10% (59). In another study in 198 localised TNBC, improved survival was observed when at least one of three classic basal-like phenotype biomarkers (including EGFR) was positive; the proposed explanation was that this was probably due to a better response to NAC (60).

Our findings are likely related to the method of scoring, while other studies were mostly binary, considering only the percentage of positivity and not the intensity of staining.

In most studies evaluating the prognostic impact of EGFR in localised breast cancer, the antibody used was Dako or Zymed, whereas we used Roche in our analysis, and positivity was considered for between 1% and 10% of stained cells, independent of intensity (56), while we used the H-score and set the cutoff according to EFS (24).

Other studies have used other scoring systems, such as one European study that analysed 52 biopsy specimens from localised triple-negative tumours before any treatment. In that study, no association was found between EGFR+ and EFS (HR 1.114, 95% CI 0.977–1.269, p = 0.106) (61). The scoring system was semiquantitative, comprising a combination of staining intensity and the percentage of positive tumour cells. Intensity was scored as 0 (no membrane staining), 1 (low), 2 (moderate), and 3 (high), and the percentage of positive cells was scored as 1 (<10%), 2 (11%–50%), 3 (51%–80%), and 4 (>80%). A final EGFR score (0–12) was then calculated by combining these two parameters (61).

Another explanation could be that in our study, the analysis was performed in the post-neoadjuvant surgical specimen, where there is significant tissue heterogeneity, and where the intensity of EGFR expression may be different from that in the primary tumour. It seems that EGFR expression decreases after NAC, and that it is not the level of expression that plays a prognostic role but rather the difference between initial expression and expression in the residual tumour (62).

In terms of Ki-67, values above 30% in the residual tumour was found to be prognostic for lower DFS (HR 3.86, 95% CI 1.19–9.21, p = 0.008) in patients receiving NAC for localised hormone receptor (HR)-HER2-negative or -positive breast cancer (63). In a larger analysis performed on residual tumours from patients previously included in the GeparTrio study (n = 1,151), of whom 58% had no pCR (n = 667) and 5.4% (n = 36) were HR-negative, Ki-67 >35% was associated with 1.73-fold increase in the risk of recurrence (95% CI 0.87–3.42) in HR-negative tumours versus Ki-67 ≤35% (64).

### Study limitations

The main limitation of this study is that due to the small number of events, we could not create a multivariate score. There is a persisting need to analyse a larger number of cases with a view to developing a prognostic score. Second, the results were interpreted in light of previous knowledge available in the literature and the prognostic value of the markers, mainly at the primary tumour level. A method for quantifying biomarker expression specific to residual cells should be developed.

## Conclusion

In this study, RD in patients with TNBC after NAC harboured mainly markers of low aggressiveness, i.e., moderate expression of basal-like biomarkers, high ARs, and low Ki-67, while markers of lymphocyte infiltration were predominantly present in the stroma. Biomarkers found to be associated with outcome and which we propose for the development of a multivariate histopathological and immunohistochemical prognostic model for EFS are AR, lymphovascular invasion (LVI), and ypN. These will be validated in a larger cohort in a subsequent study. Future research should focus on how to evaluate the evolution between primary tumour and RD by immunohistochemistry.

## Data availability statement

The raw data supporting the conclusions of this article will be made available by the authors, without undue reservation.

## Ethics statement

The studies involving humans were approved by Centre Georges Francois Leclerc, Dijon, France. The studies were conducted in accordance with the local legislation and institutional requirements. Written informed consent for participation was not required from the participants or the participants’ legal guardians/next of kin in accordance with the national legislation and institutional requirements.

## Author contributions

SI: Conceptualization, Data curation, Formal analysis, Investigation, Project administration, Resources, Supervision, Validation, Visualization, Writing – original draft, Writing – review & editing. NB: Data curation, Formal analysis, Software, Writing – original draft. GC: Data curation, Formal analysis, Software, Writing – original draft. AI: Data curation, Investigation, Resources, Visualization, Writing – original draft. FB: Investigation, Supervision, Writing – original draft. SL: Conceptualization, Resources, Writing – original draft. ID: Resources, Writing – original draft. AH: Resources, Writing – original draft. AB: Data curation, Formal analysis, Methodology, Software, Supervision, Validation, Visualization, Writing – original draft, Writing – review & editing. CC: Funding acquisition, Resources, Writing – original draft. SC: Resources, Writing – original draft. NG: Formal analysis, Investigation, Resources, Writing – original draft. BC: Resources, Writing – original draft. LA: Formal analysis, Investigation, Supervision, Validation, Visualization, Writing – original draft, Writing – review & editing.
