# Supplementary material for: Pathologic and immunohistochemical prognostic markers in residual triple-negative breast cancer after neoadjuvant chemotherapy
Source: Front Oncol. 2024 Jan 10;13:1309890. doi: 10.3389/fonc.2023.1309890 (PMC10809386; doi:10.3389/fonc.2023.1309890)
Supplement: Supplementary file 1 [file DataSheet_1.doc]

**Biomarkers immunohistochemistry protocol**

The deparaffinization was done in xylene and then slides were rehydrated in graded ethanol. The slides were brought to a temperature of 72°C. Cell conditioning and ULTRA conditioning were done by heating the slides to 95 °C and incubating for 8 minutes in CC1 (Cell Conditioning #1) buffer and incubating for 20, 36, 52 and 64 minutes in ULTRA CC1 buffer. Antibody incubation temperature was achieved by heating the slides to 36°C.

***Table: Primary monoclonal antibodies used in the present study***

| **Antigen** | **Clone** | **Supplier** | **Dilution** | **Antibody incubation time** |
| --- | --- | --- | --- | --- |
| AR | SP107, rabbit | Roche | 1:1(supplier) | 32 minutes |
| CD8 | SP57, rabbit | Roche | 1:1(supplier) | 20 minutes |
| CK5/6 | D5/16B4, mouse | Roche | 1:1(supplier) | 20 minutes |
| EGFR | D38B1, rabbit | Cell Signalling | 1:50 | 32 minutes |
| ER | SP1, rabbit | Roche | 1:1(supplier) | 24 minutes |
| FOXP3 | 236A/E7, mouse | ABCAM | 1:100 | 32 minutes |
| HER2 | 4B5, rabbit | Roche | 1:1(supplier) | 24 minutes |
| Ki67 | MIB-1, mouse | Agilent | 1:50 | 32 minutes |
| p53 | DO-7, mouse | Roche | 1:1(supplier) | 28 minutes |
| PgR | IE2, rabbit | Roche | 1:1(supplier) | 16 minutes |

Abbreviations

AR=androgen receptor, CD8=cluster of differentiation 8, CK5/6=cytokeratin 5/6, EGFR=epidermal growth factor receptor, ER=oestrogen receptor, FOXP3= fork-head box protein 3, HER2= human epidermal receptor, Ki 67= proliferation -related nuclear antigen, p53=protein 53, PgR=progesterone receptor
